# Posterior Cerebral Artery Angle and the Rupture of Basilar Tip Aneurysms

**DOI:** 10.1371/journal.pone.0110946

**Published:** 2014-10-29

**Authors:** Allen L. Ho, Amr Mouminah, Rose Du

**Affiliations:** 1 Department of Neurosurgery, Brigham and Women’s Hospital, Boston, Massachusetts, United States of America; 2 Harvard Medical School, Boston, Massachusetts, United States of America; St Michael’s Hospital, University of Toronto, Canada

## Abstract

Since the initial publication of the International Study of Unruptured Intracranial Aneurysms (ISUIA), management of unruptured intracranial aneurysms has been mainly based on the size of the aneurysm. The contribution of morphological characteristics to treatment decisions of unruptured aneurysms has not been well studied in a systematic and location specific manner. We present a large sample of basilar artery tip aneurysms (BTA) that were assessed using a diverse array of morphological variables to determine the parameters associated with ruptured aneurysms. Demographic and clinical risk factors of aneurysm rupture were obtained from chart review. CT angiograms (CTA) were evaluated with Slicer, an open source visualization and image analysis software, to generate 3-D models of the aneurysms and surrounding vascular architecture. Morphological parameters examined in each model included aneurysm volume, aspect ratio, size ratio, aneurysm angle, basilar vessel angle, basilar flow angle, and vessel to vessel angles. Univariate and multivariate analyses were performed to determine statistical significance. From 2008–2013, 54 patients with BTA aneurysms were evaluated in a single institution, and CTAs from 33 patients (15 ruptured, 18 unruptured) were available and analyzed. Aneurysms that underwent reoperation, that were associated with arteriovenous malformations, or that lacked preoperative CTA were excluded. Multivariate logistic regression revealed that a larger angle between the posterior cerebral arteries (P1-P1 angle, p = 0.037) was most strongly associated with aneurysm rupture after adjusting for other morphological variables. In this location specific study of BTA aneurysms, the larger the angle formed between posterior cerebral arteries was found to be a new morphological parameter significantly associated with ruptured BTA aneurysms. This is a physically intuitive parameter that can be measured easily and readily applied in the clinical setting.

## Introduction

Because of the availability of imaging, it has been now been determined that nearly 3% of the population has an unruptured intracranial aneurysm. [1], [2] Since the completion of the International Study of Unruptured Intracranial Aneurysms (ISUIA), the management of unruptured intracranial aneurysms has been mainly based on the size of the aneurysm. [3]–[7] However, with the recent publication of the large, prospective Unruptured Cerebral Aneurysm Study (UCAS) of Japan there has been mounting evidence for the importance not only of size, but also of the location and morphology of the aneurysm in predicting rupture risk. [8] There have been several recent aneurysm location specific studies of the contribution morphology to increased rupture risk and possible management decisions but none has specifically addressed the morphological characteristics unique to basilar tip aneurysms (BTA) [9]–[12].

Unruptured basilar tip aneurysms account for nearly 3% of all intracranial aneurysms. [1], [13] The International Subarachnoid Aneurysm Trial (ISAT) demonstrated that the basilar apex location, in addition to size, was the strongest predictor of hemorrhage. Posterior circulation location was also significantly associated with worse clinical outcome, independent of treatment modality. [14] As such, basilar artery tip aneurysms represent a unique challenge to both open surgical and endovascular interventionalists since they carry with them the greatest risk for hemorrhage, morbidity, and mortality. [14]–[16] Previous case series of basilar tip aneurysms reviewed by Nanda et. al. have focused specifically on general morphological characteristics intrinsic to the aneurysm itself that help denote ‘complex’ or more difficult to coil aneurysms including size, neck diameter, calcification/thrombosis, multi-lobes, posterior orientation, retro or sub-sellar location. We present a large sample of basilar tip aneurysms that were assessed using a diverse array of morphological variables both intrinsic to the aneurysm and related to its surrounding vasculature to determine the parameters associated with rupture.

## Methods

### Patient selection

The study population consisted of patients with basilar tip aneurysms (BTA) evaluated at the Brigham and Women’s Hospital during a 6-year period between 2008 and 2013. Exclusion criteria included aneurysms that underwent reoperation, those that were associated with arteriovenous malformations, or those that lacked preoperative CT angiography (CTA). Patient medical records were reviewed for relevant demographic and clinical information. Patient data on risk factors commonly associated with aneurysm development or aneurysm rupture were collected, including smoking status, family history, presence of multiple aneurysms, history of hypertension, and prior history of aneurysm rupture. The study was approved by the Brigham and Women’s Hospital Institutional Review Board. Patient information was anonymized and de-identified prior to analysis.

### Reconstruction of 3D models

**Figure 1:**
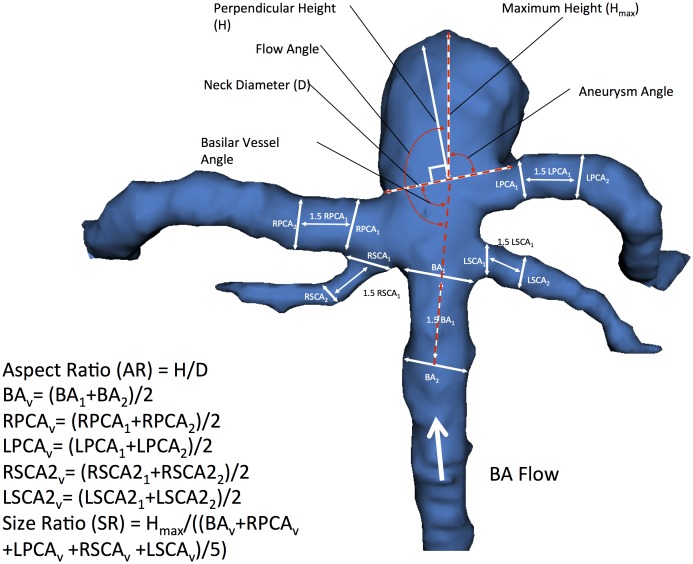
3D model of BTA aneurysm depicting morphological variables previously studied in the literature. The aspect ratio (AR) is obtained by dividing the perpendicular height by the neck diameter. Size ratio (SR) is calculated by dividing the maximum height (H_max_) by the average composite diameter of the all vessels (BA_v_, RPCA_v_, LPCA_v_, RSCA_v_, LSCA_v_) involved with the aneurysm. Composite diameters are obtained by averaging the initial diameter of the vessel (BA_1_, RPCA_1_, LPCA_1_, RSCA_1_, LSCA_1_) at the vessel branching point by the aneurysm neck with the diameter of the vessel 1.5 away from the initial diameter (BA_2_, RPCA_2_, LPCA_2_, RSCA_2_, LSCA_2_). Aneurysm angle is defined as the angle between the vectors formed by the maximum height of the aneurysm with the aneurysm neck. The vessel angle is defined as the angle between the vector of flow and the neck of the aneurysm. The flow angle is defined as the angle between the vector of flow and the vector formed by the maximum height of the aneurysm.

**Figure 2:**
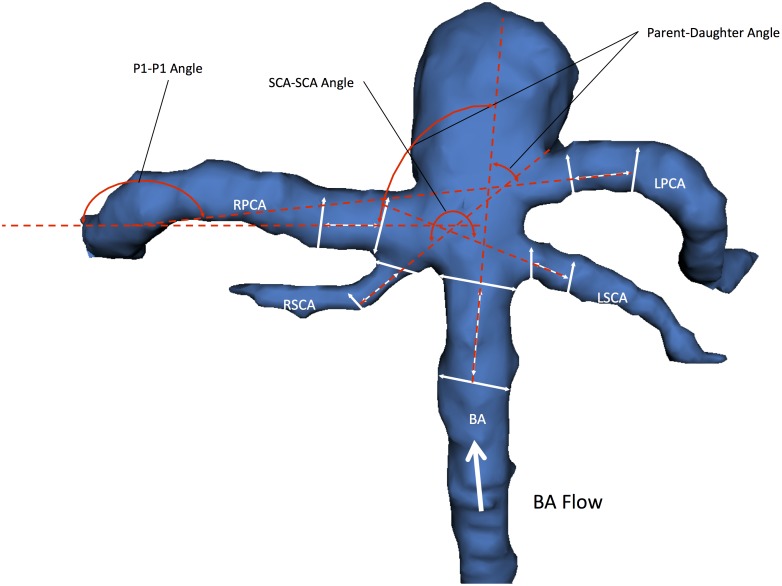
3D model of BTA aneurysm depicting angular variables of the surrounding vasculature. There were three vessel to vessel angles -measured. The Parent-Daughter angle is a composite angle that refers to the average of the two angles formed between the basilar artery (BA) and each posterior cerebral artery (RPCA, LPCA). The P1-P1 angle refers to the angle formed between the two posterior cerebral arteries (RPCA, LPCA). The SCA-SCA angle refers to the angle formed between the two superior cerebellar arteries (RSCA, LSCA).

We utilized 3D Slicer (referred as “Slicer” in the following text), an open source, multi-platform visualization and image analysis software [17], [18] as described previously [9]. Composite three-dimensional (3D) models of BTA aneurysms and their surrounding vasculature were generated with pre-operative CT angiography (CTA) images. All CTAs were performed on a Siemens SOMATOM Definition scanner with slice thickness of 0.75 mm and increment of 0.5 mm. The vascular compartment was isolated using thresholding with the brain parenchyma as reference. Aneurysm borders and contours were then reconstructed using a triangle reduction and smoothing algorithm. This 3D surface model of the aneurysm and surrounding vessels could be manipulated freely in the Slicer environment. (Figures 1 and 2) Volumes, lengths, and angles were then manually measured with fiducial-based tractography.

### Definition of morphological parameters

We examined well-studied parameters of aneurysm morphology that have been utilized in the study of aneurysms of other sub-types including aneurysm size, volume, aspect ratio, aneurysm angle, vessel angles, size ratio, flow angles, and vessel to vessel angles. [9], [19]–[21] We also defined several novel parameters that were specific to the unique anatomy of BTAs including posterior cerebral artery to posterior cerebral artery angle (P1-P1 angle), superior cerebellar artery to superior cerebellar artery angle (SCA-SCA angle). (Figures 1 and 2) Aneurysm maximum height is measured as the largest cross-sectional diameter of the aneurysm measured from the base of the aneurysm. [19], [22], [23] The maximum perpendicular height was the height of the aneurysm determined from the mid-point of the base to the dome. Aspect ratio is the ratio of the maximum perpendicular height of the aneurysm to the average neck diameter of the aneurysm. [20], [21], [24] Aneurysm angle is the angle formed between the plane of the neck of the aneurysm and the vector of the maximum height of the aneurysm. The aneurysm angle captures the angle of inclination of the aneurysm from the plane of the neck [20].

The main surrounding vessels involved with BTA aneurysms include the basilar artery proximal to the aneurysm (BA), the two superior cerebellar arteries, and the two posterior cerebral arteries bifurcating from the basilar artery and arising from the base of the aneurysm. The basilar vessel angle is angle between the basilar artery and the plane of the aneurysm neck. Vessel centerlines were determined by connecting the two center points of the vessel cross sections utilized to measure the vessel diameter in the size ratio parameter. Size ratio is defined as the ratio between the maximum aneurysm height and mean vessel diameters of all branches associated with the aneurysm. Specifically, the diameters of a particular vessel are determined by averaging the diameter of the cross section of the vessel at the vessel branching point by the neck of the aneurysm (D1) with the diameter of the cross-section at 1.5×D1 distance from the initial diameter. This average diameter was calculated for all vessels involved with the aneurysm to generate the composite mean vessel diameter utilized to calculate the size ratio [20], [25].

The basilar flow angle is the angle formed between the vector of maximum height of the aneurysm and the centerline vector through the basilar artery that represents the vector of flow. This angle captures the variation between the aneurysm angle and vessel angle, and represents the angle at which the aneurysm is tilted with respect to the vector of flow through parent vessel. [26], [27] The parent-daughter angle is a composite mean of angles formed between the centerline vector of the basilar artery with the centerline vectors of each posterior cerebral artery. With respect to a BTA, flow enters via the basilar artery and ultimately exits via the posterior cerebral arteries, and the parent-daughter angle measures the degree to which blood entering the basilar tip via the basilar artery must diverge in order to emerge into each daughter posterior cerebral artery. The P1-P1 and SCA-SCA angle are simply the angles formed between the each pair of posterior cerebral arteries and superior cerebellar arteries as measured from the direction of flow. When there is a common PCA-SCA trunk, the common origin is used as the starting point for the angle measurements. The acuity of the angle formed between these daughter vessels can have an influence on the flow dynamics encountered around the point of aneurysm formation at the basilar artery apex. Importantly, the vessel to vessel angles are independent of the aneurysm itself and capture the context of the surrounding vasculature within which the aneurysm arises.

### Statistical Analysis

Demographic and clinical characteristics were analyzed for differences by rupture status utilizing chi-square and two-tailed t-tests for binary and continuous variables, respectively. Univariate analysis was performed to compare the value of each morphological parameter between the ruptured and unruptured groups. Multivariate logistic regression was also used to calculate the odds ratios (ORs) and 95% confidence intervals (95% CI) for association with aneurysm rupture. All statistical analyses were performed using JMP Pro 10, SAS version 9.2 (SAS Institute Inc, Cary, North Carolina) and Excel 2007 (Microsoft Corp., Redmond, WA).

## Results

From 2008–2013, 54 patients with BTA were evaluated in a single institution out of which 33 CTAs were available and analyzed. All data are included in the supporting information file. There were a total of 15 ruptured and 18 unruptured aneurysms. Demographic and clinical data is provided in Table 1. The mean age was 56.5±11.9 years. Patients with ruptured aneurysms were significantly older (means of 62.73 years ruptured versus 53.89 years unruptured, p = 0.0148). There were more patients with a smoking history in the unruptured group (36% ruptured versus 64% unruptured) though this relationship only trended towards significance (p = 0.1935). There were no other major differences in clinical risk factors (gender, hypertension, presence of multiple aneurysms, family or personal history of aneurysms or previous subarachnoid hemorrhage) between the ruptured and unruptured groups.

**Table 1:** Demographic information and clinical risk factors for patients with basilar artery aneurysms.

	Unruptured (n = 18)	Ruptured (n = 15)	*p* value
Mean Age (SD)	53.89 (9.98)	62.73 (11.84)	0.0148
Female (%)	57.14	42.86	0.4095
Hypertension (%)	53.33	46.67	0.4393
Smoking (%)	63.64	36.36	0.1935
Multiple Aneurysms (%)	60	40	0.4123
Family History (%)	50	50	0.4582
Prior SAH (%)	100	0	0.1645

Univariate statistical analysis of BTA morphological parameters is also provided in Table 2. Ruptured BTA aneurysms were associated with larger maximum diameter (7.13 mm ruptured versus 6.14 mm unruptured, p = 0.2496) and volume (736.8 mm^3^ ruptured versus 422.9 mm^3^ unruptured, p = 0.1885) but these relationships were not statistically significant. Other measured variables of intrinsic aneurysm morphology, aspect ratio, aneurysm angle, and size ratio, were all very similar between ruptured and unruptured aneurysms. With regards to morphological parameters of the surrounding vasculature, there was no difference in the basilar flow angle between ruptured and unruptured groups. Ruptured aneurysms were associated with a smaller or more acute SCA-SCA angle, though this relationship was not statistically significant (221.43 ruptured versus 232.5 unruptured, p = 0.1864). BTAs in the ruptured group were associated with a larger basilar vessel angle (77.73 ruptured versus 69 unruptured, p = 0.0608) and larger parent-daughter angle (91.63 ruptured versus 80.69 unruptured, p = 0.063) in relationships that both approached statistical significance. Finally, ruptured BTAs were significantly associated with larger P1-P1 angles. This relationship was preserved in a multivariate logistic regression model that revealed that a greater P1-P1 angle was significantly associated with ruptured BTAs (OR 1.02, 95% CI 1–1.04, p = 0.037) after correcting for other morphologic parameters (Table 3).

**Table 2:** Univariate analyses for the morphological parameters measured for basilar artery aneurysms.

	Unruptured (n = 18)mean (SD)	Ruptured (n = 15)mean (SD)	*p* value
Maximum Diameter (mm)	6.14 (4.16)	7.13 (4.16)	0.2496
Aneurysm Volume (mm^3^)	422.9 (731.6)	736.8 (1172.36)	0.1885
Aspect Ratio	0.95 (0.97)	0.92 (0.42)	0.4559
Aneurysm Angle	94.31 (27.6)	97.13 (27.08)	0.3848
Size Ratio	2.69 (1.86)	2.67 (1.81)	0.4875
Basilar Vessel Angle	69 (21.29)	77.73 (8.02)	0.0608
Basilar Flow Angle	64.33 (37.3)	65.73 (39.1)	0.4587
Parent-daughter Angle	80.69 (22.32)	91.63 (17.62)	0.0630
P1-P1 Angle	145.61 (57.55)	185.13 (48.79)	0.0204
SCA-SCA Angle	232.5 (39.96)	221.43 (29.22)	0.1864
Average Basilar Artery Diameter (mm)	3.42 (0.52)	3.67 (0.68)	0.2282
Average P1 Diameter (mm)	0.45 (0.49)	0.63 (0.49)	0.1558

**Table 3:** Multivariate analyses for the morphological parameters measured for basilar artery aneurysms.

	Odds ratio (95% confidence interval)	*p* value
Neck Diameter	1.45 (0.9–2.33)	0.125
Aspect Ratio	1.51 (0.42–5.4)	0.528
Size Ratio	0.58 (0.28–1.19)	0.135
Basilar Flow Angle	1.01 (0.99–1.04)	0.272
P1-P1 Angle	1.02 (1–1.04)	0.037

## Discussion

Recent large prospective studies of intracranial aneurysms have revealed location and aneurysm morphology beyond simply size to be among the most important factors in considering aneurysm rupture risk. This has prompted the study of morphological characteristics of all different subtypes of intracranial aneurysms in a systematic and location specific manner. Studies of aneurysm morphology have been further refined to reflect three distinct determinants of the aneurysmal hemodynamics: the morphology of the aneurysm itself, the interaction between the aneurysm and the associated parent and daughter vessels, and the relationships among the surrounding vasculature. Our study examined all three of these types of morphologic variables and is the first to examine them exclusively in basilar tip aneurysms. We determined that the parameters that considered the morphology of the surrounding vasculature were most important in considering ruptured basilar artery tip aneurysms. In particular, ruptured aneurysms were most associated with a larger P1-P1 angle.

Morphological parameters beyond aneurysm size and aspect ratio were first studied in detail by Dhar et. al. who proposed a variety of variables intrinsic to the aneurysm itself as well as parameters incorporating parent vessel geometry, many of which were examined in our present study. [20] The most recent large, observation cohort study of unruptured cerebral aneurysms from Japan (UCAS) identified ‘irregularity’ of the aneurysm as defined by the presence of a daughter sac as a significant predictor of rupture risk. [8] This finding was a confirmation of earlier case-control studies that associated aneurysm rupture risk with varying definitions of ‘irregularity’. [19], [24], [28], [29] All other aneurysm intrinsic morphological variables studied including various size and shape ratios [19]–[21], [25], [26], [28], [30]–[35] and flow angles [9], [26], [29] have garnered conflicting results due to their case control design. In an effort to correct for this, a recent study by Backes et. al. compared ruptured and unruptured aneurysms in the same patients with multiple aneurysms that did find irregularity as defined by the presence of blebs, wall protrusions, or multiple lobes. [36] This remains the most consistent aneurysm intrinsic morphological variable besides size to be associated with aneurysm rupture. However, irregularity remains a subjective definition that is subject to individual user variability in evaluation. In an effort to identify quantifiable risk factors associated with rupture, including those extrinsic to the aneurysm itself, we excluded it from our present study.

Nearly every large, prospective cohort study of intracranial aneurysms has identified aneurysm location as a significant factor in determining rupture risk [5], [8], [37]–[39] Indeed, in the new PHASES aneurysm rupture risk score calculated from a pooled analysis of prospective cohort studies, location of the aneurysm is the only physical variable besides aneurysm size that is included. [40] Because of this, it stands to reason that aneurysms arising at different locations within the intracranial vasculature should be considered independently when studying variables associated with rupture. Only recently have groups begun to analyze morphological variables of aneurysms in a location specific manner similar to our present study. Previous location specific studies have demonstrated the importance of the morphology of the surrounding vasculature of an aneurysm when considering potential rupture risk. For instance, in middle cerebral artery (MCA) and anterior communicating artery (ACoA) aneurysms a sharper turn in the parent-daughter vessels (or larger parent-daughter vessel angle) was shown to be significantly associated with ruptured aneurysms. [9] In a study of posterior communicating artery (PCoA) aneurysms, a similar pattern of larger parent-daughter angles was associated with ruptured aneurysms, though this relationship was not statistically significant. [41] Similarly, our study of BTAs and their surrounding vasculature revealed that ruptured aneurysms were associated with larger parent to daughter angle in a relationship that approached significance. As discussed in our previous studies, the parent to daughter vessel angle is a representation of the deviation of blood flow from the parent basilar artery into the daughter posterior cerebral arteries. Ruptured BTAs were also nearly significantly associated with larger basilar vessel angles and were significantly associated with larger P1-P1 angles in a relationship that was preserved in multivariate analysis. A larger P1-P1 angle also represents a greater divergence of flow from the originating basilar artery into both of the daughter PCA vessels. Anatomically, BTAs are located at the bifurcation point of the basilar artery into the PCAs and the effect the parent to daughter and P1-P1 angles have on aneurysm formation and rupture risk can be conceptualized via the hemodynamics of a vascular bifurcation point. Fluid flow and wall shear stress at and near the bifurcation point of a vessel can be modeled using various three-dimensional fluid mechanics model of flow and have demonstrated that a larger bifurcation angle leads to lower wall shear stress. [42]–[45] Lower wall shear stress has been linked to endothelial cell dysfunction [46]–[48], as well as aneurysm origination and rupture. [46], [49]–[51] In this way, a larger parent to daughter and P1-P1 angle represent a larger bifurcation angle that may lead to lower wall shear stress and increased risk of rupture in our study BTAs, though this is a relationship needs to be confirmed with further study and hemodynamic modeling.

### Limitations

The main limitations to this, and other similar studies, are related to the retrospective case-control study design. The case control study design may lead to confounding by patient specific characteristics, although we did study these in our patient population and only age and smoking were different between groups. All inferences made about the parameters examined can be associated with ruptured aneurysms only, and are not necessarily predictors of rupture risk. Furthermore, certain features of ruptured aneurysm may have been altered by the ruptured state. Nevertheless, the significant parameters in this study are largely that of the surrounding vasculature, which would not be altered by the rupture of an aneurysm. In addition, measurements were performed manually with the 3D Slicer software. Other groups have begun to devise more automated methods of assessing morphology to achieve greater consistency [52], but we believe that the method of our analysis best represents the applicability of our methodology in a clinical setting. Moreover, the use of simplified morphological variables does not explain the fluid dynamics or mechanisms underlying aneurysm growth and rupture. This will need to be examined in a future study that examines the fluid dynamics in detail. However, the simplification affords the clinician the ability to make measurements utilizing patient CTAs and open source software in an efficient manner. Finally, given our small sample size, our findings will need to be validated by larger prospective follow-up studies.

## Conclusions

We conducted a dedicated study of the morphological characteristics of basilar tip aneurysms and found that ruptured aneurysms were associated with larger basilar vessel angle and larger parent to daughter angle in relationships that approached significance, and were significantly associated with larger P1-P1 angles in a relationship that was preserved in multivariate analysis. Though these variables do not replace well-established clinical determinants of BTA rupture risk, these features do add to the growing body of evidence that surrounding vasculature characteristics may have a significant effect on aneurysm hemodynamics that could influence rupture risk. Careful consideration of the surrounding vasculature is a technique that can be rapidly applied by clinicians when examining 3D reconstructions of unruptured aneurysms. Furthermore, the P1-P1 angle is unique to the BTAs and highlights the importance of location specific features when determining the natural history of aneurysms.

## Supporting Information

Data S1 (CSV)
